# Study on UV Aging of Thermoplastic Polyurethane and Its Crosslinked Product

**DOI:** 10.3390/polym18141778

**Published:** 2026-07-21

**Authors:** Hanyang Zhao, Qingjun Jin, Hongwei Zhao, Yunkai Yang, Xiang Cheng, Xiujuan Ren, Hongxing Shi

**Affiliations:** Chemical Defense Institute, Academy of Military Sciences, Beijing 102205, China; zhy17810287009@163.com (H.Z.); jinqingjun502@163.com (Q.J.); zhw211@126.com (H.Z.); aubrey_sir@163.com (Y.Y.); 1229136@mail.dhu.edu.cn (X.C.)

**Keywords:** thermoplastic polyurethane, UV aging, glass transition, crosslinked product

## Abstract

To elucidate the formation of crosslinked products and their influence on material degradation, thermoplastic polyurethane (TPU) films were subjected to accelerated UV aging for various durations. Post-aging, the samples underwent Soxhlet extraction with tetrahydrofuran (THF), yielding an insoluble fraction—operationally defined as the crosslinked product—and a soluble uncrosslinked fraction. The mechanical properties, molecular weight distribution, swelling behavior, thermal properties, and chemical structure were analyzed. As UV aging progressed, both tensile strength and elongation at break deteriorated markedly. Concurrently, GPC analysis revealed a continuous decrease in molecular weight and a broadening of the molecular weight distribution, confirming that chain scission was the dominant degradation pathway. An insoluble network-like residue, defined as the crosslinked product, first appeared after 12 h of aging, with its content increasing to 22.9% after 300 h. Swelling tests showed that the crosslinked product had a high gel fraction, and its swelling ratio decreased from 196.8% to 157.4%, indicating the formation of a stable and increasingly dense network. DSC and TG results revealed restricted segmental motion, altered thermal transition behavior, and enhanced char-forming ability. The glass transition temperature of the crosslinked product exceeded that of the pristine TPU film. FTIR analysis showed variations in the -NH_2_, C=O, C-O, and C-O-C bands, confirming structural evolution within both hard and soft segments. In summary, UV aging of TPU involves a complex interplay among chain scission, degradation of soft segments, rearrangement of hard segments, evolution of hydrogen bonds, and radical-induced crosslinking. Crucially, the crosslinked network formed during aging plays a pivotal role in determining the macroscopic structural, thermal, and mechanical properties of the polymer.

## 1. Introduction

Thermoplastic polyurethane (TPU) elastomers have been widely used in engineering applications because of their excellent solvent resistance, abrasion resistance, and mechanical properties [1,2,3]. However, long-term exposure to sunlight renders TPU susceptible to UV-induced aging, leading to the deterioration of its material properties. Therefore, it is essential to investigate the UV-aging behavior of TPU [4,5,6], evaluate the effects of aging products on material performance, and elucidate the structural and property evolution during aging [7,8,9,10,11].

The photo-oxidative degradation of TPU under UV irradiation is governed by complex mechanisms. Pioneering work by Gardette and Lemaire [12] on MDI-based TPU identified two primary photochemical pathways: Process A, involving the photo-oxidation of the MDI moiety to form yellowing-inducing quinone-imide chromophores, and Process B, involving the photo-Fries rearrangement of urethane linkages, which may partially retard photo-oxidation.

Beyond chromophore formation, the direct photolytic cleavage of chemical bonds represents another critical degradation pathway. Specifically, the scission of C-N and C-O bonds within urethane linkages [13] not only triggers macromolecular degradation but also generates reactive free radicals, which subsequently undergo recombination to yield crosslinked architectures [14].

Prater [15] investigated the UV aging process of TPU and found that the oxidative cleavage of ether segments produces methylene groups or directly generates carboxylic acid groups. Hoyle and Bajsic [16,17] reported that because TPU contains both soft and hard segments, the effect of UV irradiation depends on their relative contents; typically, a higher hard-segment content increases crystallinity, rendering the material less susceptible to UV aging. Wilhelm et al. [18] studied the UV aging mechanism of polyether-based polyurethane, revealing that ether bond oxidation occurs in several stages. Initial oxidation forms unstable intermediate radicals, which subsequently form ester compounds and water, with some intermediates further reacting to form carboxylic acids and alcohols. Aglan and Ludwick [19,20] investigated the dynamic thermomechanical properties of TPU after UV irradiation, finding that UV aging increases both the storage modulus and the glass transition temperature.

The TPU film used in this work is a commercial polyether-based thermoplastic polyurethane with robust mechanical properties and broad engineering applicability. Since such materials are frequently exposed to sunlight and hygrothermal environments during service, understanding their UV-aging behavior is essential for evaluating service durability and improving weatherability.

Despite these extensive studies, the precise nature, structural evolution, and specific role of the crosslinked products generated during TPU photo-aging remain largely elusive. To bridge this knowledge gap, the present study systematically investigated the time-dependent degradation behavior of TPU films subjected to accelerated UV aging. Specifically, the macroscopic mechanical performance and underlying molecular weight distributions were monitored as a function of aging duration (0–300 h) using tensile testing and GPC. Furthermore, a THF-extraction protocol was employed to isolate the yellow, network-like crosslinked fraction, enabling comprehensive characterization to elucidate its structural evolution and contribution to the aging mechanism of TPU.

## 2. Materials and Methods

**Figure 1:**
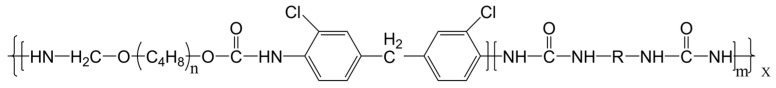
Chemical structure of the TPU used in this study.

Materials: Thermoplastic polyurethane (TPU) films (grade 8185RV, 0.2 mm thickness) were supplied by Wanhua Chemical Group Co., Ltd. (Yantai, China). Analytical-grade anhydrous ethanol and chromatographic-grade tetrahydrofuran (THF) were purchased from Shanghai Aladdin Biochemical Technology Co., Ltd. (Shanghai, China). The chemical structure of the TPU used in this study is shown in Figure 1.

Preparation of TPU films and UV aging treatment: Rectangular TPU specimens (7.5 cm × 15 cm) were prepared, cleaned with anhydrous ethanol, vacuum-dried, and subsequently stored in an amber desiccator prior to aging. Accelerated UV aging was conducted in accordance with ISO 4892-3 [21] utilizing a climate chamber equipped with UVB-313 fluorescent lamps. The specimens were subjected to cyclic aging conditions consisting of 8 h of UV irradiation at 70 °C with an irradiance of 0.48 W/m^2^, followed by 4 h of dark condensation at 50 °C. To capture the time-dependent degradation kinetics, samples were retrieved at predetermined cumulative exposure intervals (specifically 2, 6, 12, 25, 36, 50, 100, and 300 h) and stored in the dark prior to subsequent characterizations. This protocol primarily simulates UV-dominated photo-oxidative aging, wherein the elevated temperature (70 °C) serves as an accelerating factor to expedite the degradation process.

**Figure 2:**
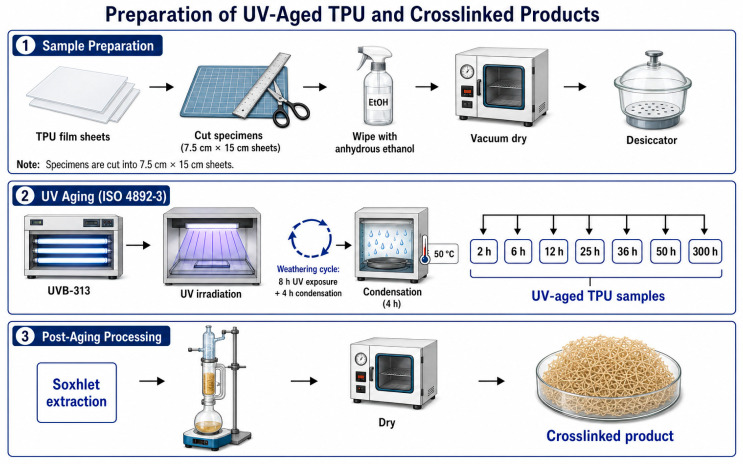
Schematic illustration of the preparation of the crosslinked product by Soxhlet extraction.

Preparation of the crosslinked product by Soxhlet extraction: To isolate the crosslinked fraction, aged TPU films were placed in cellulose thimbles and subjected to continuous Soxhlet extraction with THF. The reflux process was maintained until the solvent remained colorless, ensuring the exhaustive removal of soluble oligomers and uncrosslinked chains. The resulting THF-insoluble residue was recovered, vacuum-dried at ambient temperature to a constant mass, and designated as the crosslinked product. A schematic illustration of the preparation method is shown in Figure 2.

In this study, the term “crosslinked product” is used to operationally define the THF-insoluble fraction. Its formation is inferred from macroscopic and indirect evidence (e.g., insolubility and network morphology) rather than being directly verified by quantitative chemical crosslink density measurements.

Characterization methods: Mechanical properties were evaluated according to the GB/T 528-2009 standard [22]. UV-aged TPU films (0.2 mm thick) were cut into Type I dumbbell-shaped specimens. Tensile strength and elongation at break were measured using an AI-7000M-GD electronic universal testing machine (Gotech Testing Machines, Dongguan, China) at a crosshead speed of 500 mm/min. Five replicates were tested for each sample to ensure statistical significance.

FTIR spectra were recorded using a Nicolet 6700 FTIR spectrometer (Thermo Fisher Scientific, Waltham, MA, USA) equipped with an attenuated total reflectance (ATR) accessory (ZnSe crystal, 42° incident angle, 2.03 μm penetration depth at 1000 cm^−1^). Spectra were collected in the range of 4000–800 cm^−1^ using 32 scans at a resolution of 4 cm^−1^.

Molecular weight distributions were determined using an Alliance gel permeation chromatography (GPC) system (Waters Corporation, Milford, MA, USA). Sample solutions (1.0–1.5 mg/mL) were injected three times (injection volume: 20 μL per injection).

Differential scanning calorimetry (DSC) was performed using a DSC 25 (TA Instruments, New Castle, DE, USA) under a nitrogen atmosphere. Samples (5–10 mg) were heated from −70 °C to 220 °C, cooled to −70 °C, and reheated to 220 °C at a heating rate of 10 °C/min, with a 3 min isothermal hold at each stage.

Swelling experiments were conducted by immersing dried samples (initial mass m_0_) in THF at room temperature for 48 h to reach swelling equilibrium. The swollen samples were blotted with filter paper to remove excess surface solvent and weighed immediately (m_s_) using a Mettler Toledo ME204 analytical balance (Mettler Toledo, Greifensee, Switzerland; max capacity: 220 g, readability: 0.1 mg, repeatability: 0.08 mg). Subsequently, they were vacuum-dried at 60 °C to constant weight (m_d_). The swelling ratio and gel fraction were calculated as (m_s_ − m_d_)/m_d_ × 100% and m_d_/m_0_ × 100%, respectively, with each measurement performed in triplicate and the mean value reported.

## 3. Results

### 3.1. Characterization Results of TPU Films

#### 3.1.1. Mechanical Properties of TPU Films

**Figure 3:**
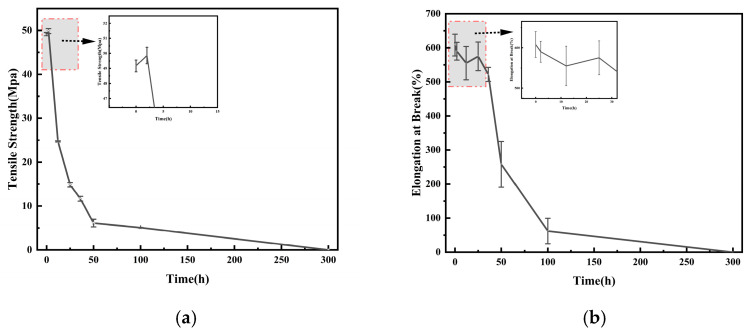
Mechanical properties of TPU films after different UV-aging times: (**a**) tensile strength and (**b**) elongation at break.

Figure 3 depicts the evolution of the mechanical properties of TPU films subjected to UV irradiation over durations ranging from 0 to 300 h. Relative to the pristine sample, a slight increment in tensile strength was observed after 2 h of aging, accompanied by a concurrent decline in elongation at break. This initial divergence is plausibly attributed to early-stage structural rearrangements or localized physical aging phenomena. With prolonged aging, both tensile strength and elongation at break exhibited an overall decreasing trend [23].

Between 2 and 36 h, the mechanical properties gradually deteriorated, implying that UV irradiation induced chemical bond cleavage and partial structural damage. After 50 h, the properties declined rapidly; by 100 h, the tensile strength and elongation at break dropped to approximately 5 MPa and 62%, respectively. These results indicate that prolonged UV irradiation severely damaged the internal TPU structure, leading to a significant loss in macroscopic mechanical properties.

#### 3.1.2. FTIR Analysis Results of TPU Films

**Figure 4:**
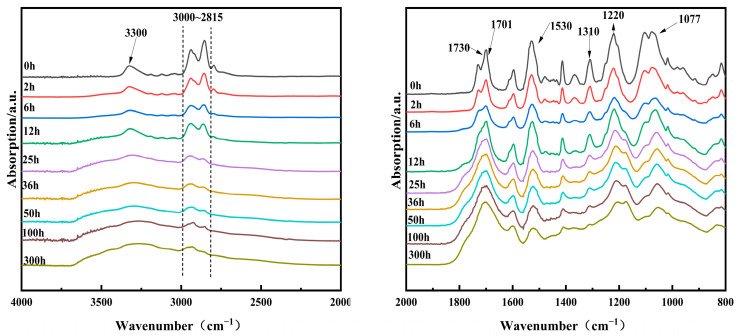
FTIR spectra of TPU films subjected to different UV aging times.

Figure 4 presents the FTIR spectra of TPU films after various UV aging times. The unaged film exhibited typical polyurethane bands: N-H stretching of urethane groups near 3330 cm^−1^, C-H stretching vibrations of methyl and methylene groups in the range 2815–3000 cm^−1^. Urethane carbonyl (C=O) absorption occurred in the region 1680–1750 cm^−1^, and amide II and III bands appeared near 1530 and 1310 cm^−1^, respectively. Additionally, the band near 1220 cm^−1^ was attributed to C-O stretching in hard-segment urethane groups, while bands at 1104 and 1077 cm^−1^ corresponded to C-O-C stretching of ether bonds in the polyether soft segments [24].

Upon UV exposure, the FTIR spectra exhibited profound alterations in characteristic absorption profiles. Notably, within the initial 6 h, the intensities of the N-H stretching (3330 cm^−1^) and alkyl C-H stretching bands in the region 2815–3000 cm^−1^ decreased markedly, indicating MDI hard-segment degradation.

After 12 h, new absorption bands emerged near 3445 cm^−1^ and 3245 cm^−1^, indicative of the formation of new structural features during prolonged UV exposure.

In the carbonyl region, the band near 1730 cm^−1^ in the unaged sample was assigned to free carbonyl groups, while the band near 1700 cm^−1^ corresponded to hydrogen-bonded carbonyls [25], indicating the presence of physical crosslinking points. After 12 h, the band at 1701 cm^−1^ dominated the carbonyl region, and overall carbonyl intensity gradually decreased with aging time. Concurrently, the urethane C-O (1220 cm^−1^) and ether C-O-C (1104 and 1077 cm^−1^) bands weakened, suggesting molecular chain degradation under UV irradiation. Beyond 50 h, the C-H stretching bands in the range 2815–3000 cm^−1^ nearly disappeared, suggesting severe chain scission, possibly accompanied by the formation of CO_2_ and other low-molecular-weight products.

#### 3.1.3. GPC Analysis of TPU Films

**Figure 5:**
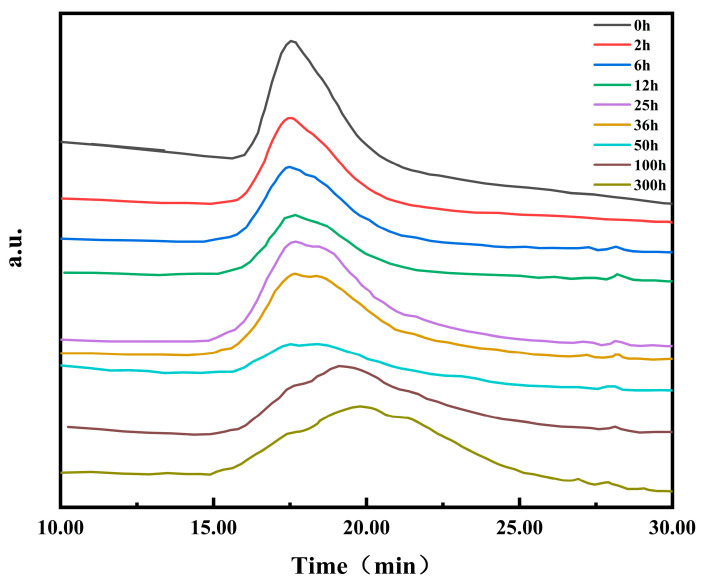
GPC results of TPU samples after UV aging for different times.

GPC analysis corroborated the progressive photolytic scission of TPU macromolecules, evidenced by a substantial reduction in average molecular weight as a function of aging time. Intriguingly, at the 2 h mark, the number-average molecular weight (Mn) slightly exceeded that of the unaged sample, which may be associated with early-stage variations in low-molecular-weight species, consistent with the temporary tensile strength increase. With prolonged aging, the molecular weight continuously decreased in parallel with the decline in mechanical properties, confirming that extended UV aging degrades the TPU molecular structure. The molecular weight distribution curve is shown in Figure 5.

Additionally, the polydispersity index (PDI) gradually increased with aging time (Table 1), indicating a broader molecular weight distribution during UV aging. This indicates that TPU chains were degraded to different extents, gradually forming short-chain fragments and low-molecular-weight degradation products, further confirming UV-induced structural damage.

### 3.2. Characterization Results of Crosslinked Product

#### 3.2.1. Crosslinked Product Content and Swelling Behavior

**Figure 6:**
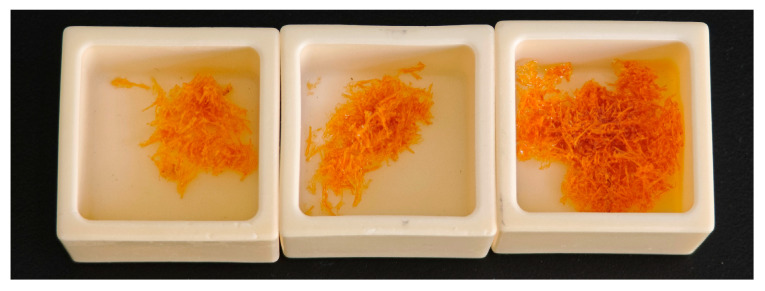
Crosslinked product of TPU after UV aging.

Aged TPU films were subjected to Soxhlet extraction using THF. No discernible precipitate was observed for samples aged for 0, 2, and 6 h. Conversely, samples aged for 12, 25, 36, 50, 100, and 300 h yielded a yellow, flocculent, insoluble network-like precipitate. This precipitate, undissolved during extraction, was collected after washing and drying, and designated as the crosslinked product. This designation relies on indirect evidence (THF insolubility, network morphology, high gel fraction, and reduced swelling ratio), which aligns with the formation of a physical or chemical network rather than directly proving exclusive covalent crosslinking. The THF-soluble fraction was defined as the uncrosslinked product. The crosslinked product obtained by Soxhlet extraction is presented in Figure 6.

As shown in Table 2, the mass fraction of the crosslinked product increased non-linearly with aging time. It rose rapidly within the first 36 h, but its growth rate slowed significantly thereafter. This may be attributed to the competition between the formation of new crosslinked structures and the photodegradation of existing ones; this competition reduced the overall mass growth rate.

Swelling results (Table 3) show that the gel fraction exceeded 97% across all aging times, indicating high THF insolubility and a stable network. From 12 to 300 h, the gel fraction increased from 97.7% to 99.4%, while the swelling ratio decreased from 196.8% to 157.4%. This reduction in solvent-induced expansion implies that the network became increasingly dense, thereby restricting chain mobility as aging proceeded.

Notably, between 50 and 100 h, the swelling ratio decreased marginally (173.1% to 171.1%), whereas the gel fraction increased from 97.3% to 98.8%. The 300 h-aged sample exhibited the highest gel fraction and lowest swelling ratio, indicating the most complete and stable network. Overall, these results indirectly support the formation of an increasingly compact, insoluble network-like structure during UV aging, consistent with chemical crosslinking.

#### 3.2.2. DSC Analysis of the Crosslinked Product

**Figure 7:**
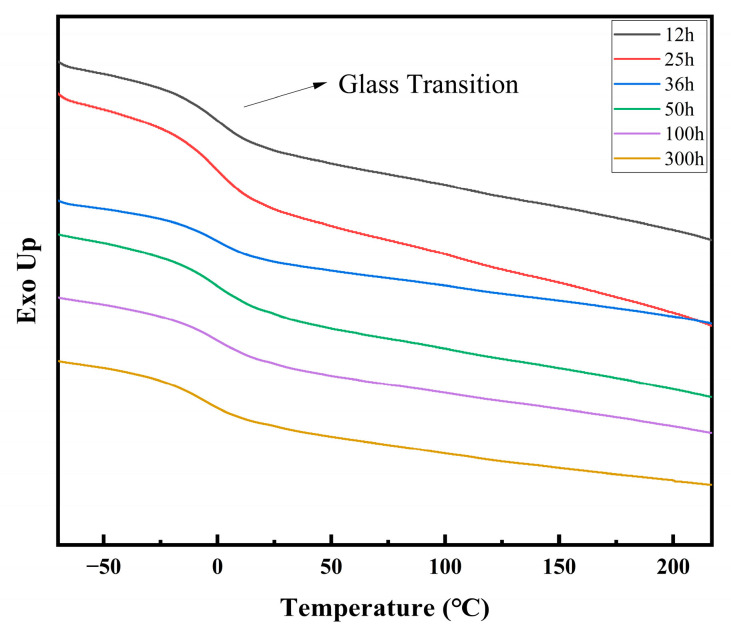
DSC curves of crosslinked product.

DSC measurements of the crosslinked product (Figure 7, Table 4) showed no distinct endothermic melting peaks below 220 °C, suggesting a lack of apparent melting behavior. Combined with its THF insolubility, this is consistent with the typical infusible and insoluble characteristics of crosslinked polymers, further justifying its designation based on indirect evidence.

**Figure 8:**
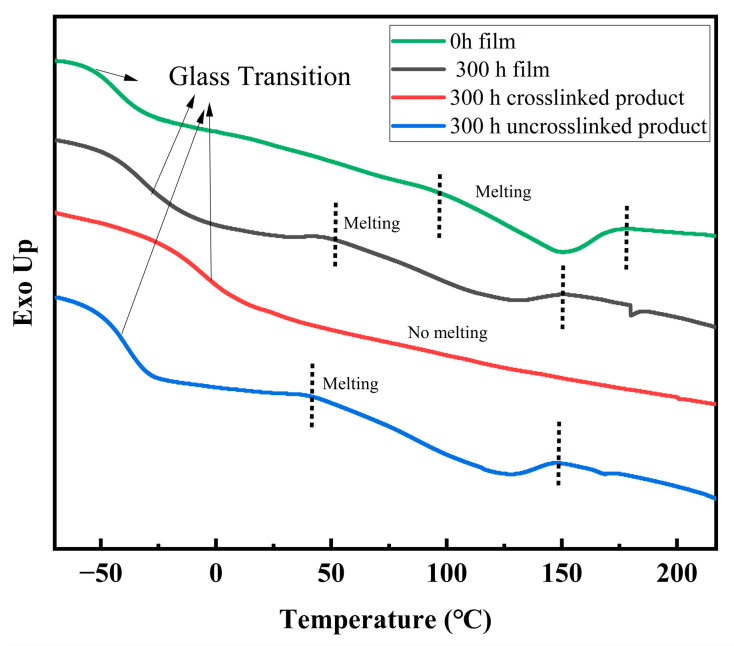
DSC curves of each component after aging for 300 h.

The glass transition temperatures (T_g_) of the crosslinked products remained around −10 °C, with little variation among samples aged for different times, significantly exceeding that of unaged TPU. As shown in Figure 8 and Table 5, the T_g_ of the unaged film was −47 °C, increasing to −34 °C after 300 h of aging. In the 300 h-aged sample, the T_g_ of the uncrosslinked product was −41 °C, whereas that of the crosslinked product reached −12 °C. This implies that the crosslinked structure strongly restricts segmental motion, which serves as a key factor contributing to the overall T_g_ increase in aged TPU.

The 0 h film exhibited a melting endotherm at 150 °C, associated with the melting of ordered hard-segment domains, indicating that the pristine film contained relatively well-developed hard-segment organization. After 300 h, this peak weakened and shifted to a lower temperature, suggesting partial reduction in hard-segment order during aging. The uncrosslinked product retained a clear melting peak, indicating residual crystallization ability. Conversely, the crosslinked product lacked a distinct melting peak, indicating that the network structure hinders chain rearrangement and suppresses the formation of ordered domains capable of melting.

#### 3.2.3. TG Analysis of the Crosslinked Product

**Figure 9:**
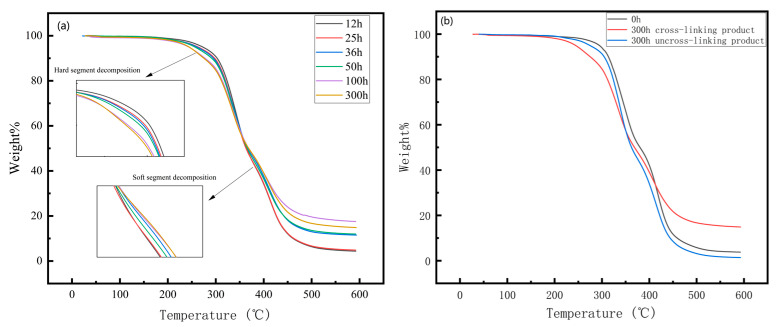
TG curves (**a**) TG of crosslinked product obtained at different aging times; (**b**) different fractions obtained after 300 h of UV aging.

Thermogravimetric analysis of the crosslinked product under nitrogen revealed two main decomposition stages. The lower-temperature stage (~300 °C) was mainly associated with the cleavage of urethane bonds and hard-segment structures, while the higher-temperature stage (~400 °C) was attributed to soft-segment decomposition. With increasing aging time, the characteristic temperature of the lower-temperature stage decreased, indicating hard-segment damage, whereas the higher-temperature stage shifted to higher temperatures. The results of TG are shown in Figure 9.

A comparative analysis was further conducted among the unaged film, the 300 h-aged crosslinked product, and the 300 h-aged uncrosslinked product. The unaged TPU exhibited generally higher decomposition temperatures than the uncrosslinked product, indicating reduced thermal stability after prolonged UV irradiation. The crosslinked product exhibited a higher final char residue than the uncrosslinked product, pointing to an enhanced char-forming ability. This may be attributed to network formation, enrichment of carbonaceous structures, and cleavage of oxygen- or nitrogen-containing groups, which release volatile molecules during UV aging. Thus, TG results further confirm complex chemical changes in TPU during UV aging.

#### 3.2.4. FTIR Analysis of the Crosslinked Product

**Figure 10:**
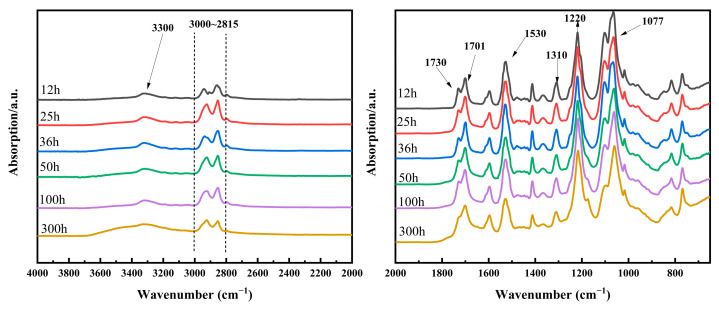
FTIR spectra of the crosslinked product.

FTIR spectra of the crosslinked product (Figure 10) were normalized, and characteristic peak areas were integrated (Table 6). Compared to the TPU films, the crosslinked product exhibited a markedly broadened -NH stretching band (3326–3330 cm^−1^) with an increased relative area. This indicates a higher proportion of hard-segment structures, suggesting that the –NH groups are involved in more complex hydrogen bonding and chemical environments.

The carbonyl band near 1730 cm^−1^ in the crosslinked product consistently displayed a clear double-peak feature, which weakened or disappeared in the TPU films after 12 h. This indicates distinct carbonyl structures and hydrogen-bonding states in the crosslinked product compared to the film matrix. The relative areas of the 1730 cm^−1^ carbonyl and 1220 cm^−1^ urethane C-O bands fluctuated rather than increasing continuously, reflecting ongoing chain cleavage and rearrangement, consistent with mechanical deterioration. Additionally, the decreased relative area of the ether C-O-C band (1066 cm^−1^) indicates significant polyether soft-segment degradation during UV irradiation.

**Figure 11:**
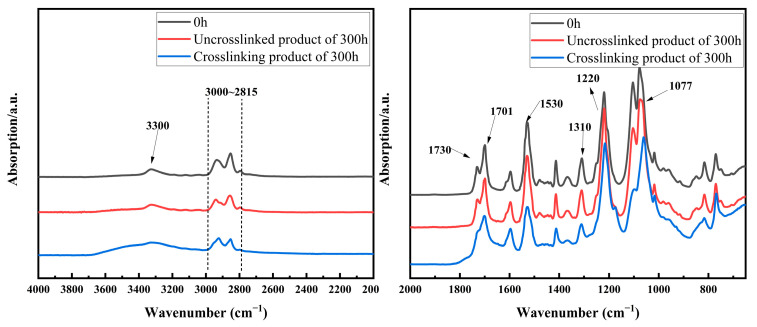
FTIR spectra of the product obtained after 300 h of UV aging.

**Figure 12:**
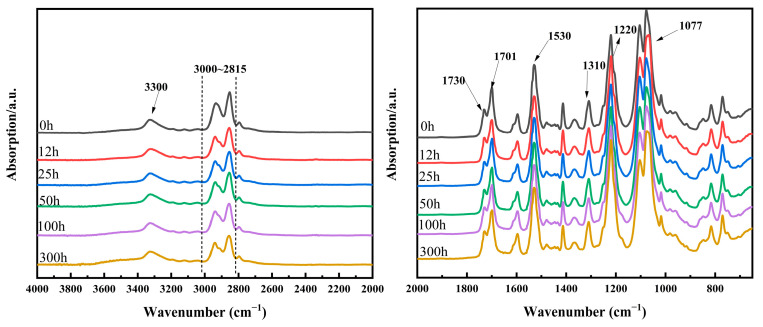
FTIR spectra of the uncrosslinked product obtained after different UV aging times.

The FTIR spectra of the different fractions after 300 h of UV aging are shown in Figure 11. The FTIR spectra of the uncrosslinked fractions obtained after different aging times are shown in Figure 12.

The carbonyl bands near 1701 cm^−1^ and 1730 cm^−1^ were deconvoluted using a Lorentz function to calculate the hydrogen-bonding index (HBA) (Table 7). The HBA of the crosslinked product increased during the first 50 h, indicating a higher proportion of hydrogen-bonded carbonyls, but decreased beyond 50 h, reflecting further structural changes in carbonyl-containing groups during prolonged aging.

(1) $$\mathrm{HBA}=\frac{A1701}{A1701+A1730}\times 100\%$$

Hydrogen bonds serve as crucial physical crosslinking points in TPU, strongly influencing phase separation, crystallization, and mechanical properties. A decrease in hydrogen-bond content weakens the restriction on soft-segment motion [26], thereby increasing chain mobility and potentially promoting phase separation or aggregation.

## 4. Discussion

The mechanical evaluations underscore a pronounced time-dependent deterioration of TPU films induced by UV aging. Although the slight initial increase in tensile strength at 2 h could be attributed to early-stage structural rearrangements or incipient localized crosslinking, the simultaneous decline in elongation at break signifies a compromise in material flexibility, implying restricted soft-segment motion or incipient chain damage even during short-term exposure.

With prolonged irradiation, both tensile strength and elongation at break decreased, suggesting that chain scission became the dominant degradation mechanism. Molecular chain cleavage reduces effective chain length and weakens the load-bearing network, diminishing resistance to stress and deformation. The rapid mechanical decline after 50 h confirms severe structural damage from extended UV exposure. By 100 h, the extremely low strength and elongation indicate a substantial loss of mechanical integrity. The mechanical deterioration is thus primarily attributed to progressive molecular destruction during UV aging.

GPC results confirm that UV irradiation progressively cleaves macromolecules into short-chain fragments and low-molecular-weight degradation products, aligning with the mechanical decline. FTIR results further demonstrate that both soft and hard segments participate in UV-aging reactions, evidenced by significant changes in the -NH, C=O, C-O, and C-O-C characteristic bands.

The manifestation of the yellow crosslinked network after 12 h provides compelling evidence that UV-generated macroradicals undergo recombination and crosslinking reactions concurrently with predominant chain scission. Although the mass fraction of this crosslinked product exhibits a monotonic increase over time, the decelerating growth kinetics observed at later stages strongly imply a dynamic competition between the formation of nascent crosslinks and their subsequent photolytic degradation.

Thermal analysis via DSC reveals that the isolated crosslinked product possesses a substantially elevated glass transition temperature (T_g_) relative to both the pristine film and the uncrosslinked fraction. This marked shift to higher Tg values is highly consistent with the severe restriction of segmental mobility imposed by the formation of a robust, three-dimensional network structure within the insoluble fraction. Consequently, the development of crosslinked structures emerges as a critical factor contributing to the overall T_g_ increase in aged TPU. Furthermore, the melting endotherm observed in the DSC curves reflects UV-induced microphase structural changes. The broadening, weakening, or shift in this endothermic peak in the aged film and uncrosslinked product indicates that chain scission and oxidation disrupt hard-segment packing and ordered hydrogen-bonded structures. Conversely, the absence of a distinct melting peak in the crosslinked product implies that the network may restrict chain diffusion and ordered domain formation. These thermal transition changes align with FTIR results, collectively substantiating the premise that UV aging alters hydrogen bonding, urethane linkages, and ether bonds.

FTIR results confirm structural evolution during UV aging. The broadened -NH band and its increased relative area in the crosslinked product indicate an enrichment of hard-segment structures and a more complex hydrogen-bonding environment. This correlation suggests that hard-segment rearrangement and changes in hydrogen bonding are linked to the formation of crosslinked products. The persistent double-peak carbonyl feature in the crosslinked product, which weakened in aged films, highlights distinct carbonyl environments and hydrogen-bonding structures compared to the film matrix. Furthermore, variations in the carbonyl and urethane C-O bands reflect continuous cleavage and rearrangement during aging.

The decrease in the intensity of the ether C-O-C band confirms polyether soft-segment degradation, which weakens the flexible phase and aligns with the mechanical decline. HBA results further reveal that the proportion of hydrogen-bonded carbonyls increased before 50 h and subsequently decreased, reflecting ongoing structural modifications. Overall, the FTIR results demonstrate that UV aging of TPU involves chain scission, hard-segment rearrangement, degradation of soft segments, evolution of hydrogen bonding, and crosslinking.

## 5. Conclusions

In this study, TPU films were subjected to accelerated UV aging to systematically investigate the structural evolution and properties of the aged films, as well as the derived crosslinked and uncrosslinked products. The main conclusions are summarized as follows:

Accelerated UV aging induced severe, time-dependent mechanical deterioration in TPU films. Despite a transient slight increase in tensile strength during the first 2 h, prolonged irradiation led to a drastic decline in both strength and elongation at break, reflecting progressive degradation of the macromolecules.

GPC analysis revealed a continuous decrease in molecular weight accompanied by a broadening of the molecular weight distribution, confirming ongoing chain scission and the generation of short-chain fragments and low-molecular-weight degradation products.

A yellow, network-like insoluble residue (defined as the crosslinked product) emerged after 12 h, signifying network development. Its mass fraction increased over time, albeit at a decelerating rate. Swelling analysis demonstrated a high gel fraction and a decreasing swelling ratio, indirectly corroborating the formation of a stable, increasingly dense network during UV aging.

DSC results revealed a significantly higher glass transition temperature for the crosslinked product compared to both the unaged film and the uncrosslinked product, implying a strong restriction of segmental motion. Furthermore, the absence of a distinct melting peak indicated that the crosslinked network disrupted the ordered hard-segment structure.

TG analysis showed two primary decomposition stages for the crosslinked product, with significantly altered thermal behavior compared to that of the unaged TPU. The increased residual char indicated enhanced char-forming ability, further confirming complex chemical transformations during UV aging.

FTIR analysis revealed distinct variations in the -NH, C=O, C-O, and C-O-C bands. The increased -NH band area and the persistent carbonyl double-peak indicated altered hard-segment structures and hydrogen-bonding states, while the diminished ether C-O-C band confirmed the severe degradation of polyether soft segments.

In conclusion, the UV photo-aging of TPU constitutes a highly complex physicochemical process governed by the intricate interplay between chain scission, soft-segment degradation, hard-segment rearrangement, hydrogen-bond disruption, and radical-mediated crosslinking. Crucially, the formation and accumulation of the crosslinked network play a pivotal role in determining the macroscopic structural, thermal, and mechanical evolution of TPU during UV exposure.

## Figures and Tables

**Table 1:** Molecular weights of TPU samples after UV aging for different times.

UV Aging Time	Mw	Mn	PDI
0	110,900	76,899	1.44
2	110,693	77,067	1.44
6	105,244	63,704	1.65
12	100,343	62,916	1.55
25	95,156	45,556	2.09
36	87,004	32,614	2.67
50	89,933	41,673	2.16
100	58,513	16,254	3.60
300	49,304	13,445	3.67

**Table 2:** Crosslinked product contents of samples subjected to different UV-aging durations.

UV Aging Time	Crosslinked Product Content
0	0
2	0
6	0
12	9.5%
25	13.0%
50	17.0%
100	20.0%
300	22.9%

**Table 3:** Results of the swelling test.

Sample	m_0_/mg	m_s_/mg	m_d_/mg	Swelling Ratio	Gel Fraction
Crosslinked product-12	129	374	126	196.8%	97.7%
Crosslinked product-25	134	367	130	182.3%	97.0%
Crosslinked product-50	149	396	145	173.1%	97.3%
Crosslinked product-100	161	431	159	171.1%	98.8%
Crosslinked product-300	177	453	176	157.4%	99.4%

**Table 4:** Glass transition temperature of crosslinked product.

Aging Time	Glass Transition Temperature
12 h	−11 °C
25 h	−12 °C
36 h	−10 °C
50 h	−11 °C
100 h	−8 °C
300 h	−12 °C

**Table 5:** Glass transition temperatures of the products obtained after 300 h of UV aging.

Sample Name	Glass Transition Temperature
UV aging 0 h film	−47 °C
UV aging 300 h film	−34 °C
UV aging 300 h crosslinked product	−12 °C
Uncrosslinked product after 300 h UV aging	−41 °C

**Table 6:** Relative areas of the main characteristic peaks of the crosslinked and uncrosslinked products.

Aging Time (Crosslinked/Uncrosslinked)	0 h	12 h	25 h	50 h	100 h	300 h
3324 cm^−1^-NH-, -NH_2_	0.079	0.094/0.074	0.091/0.078	0.114/0.079	0.097/0.089	0.163/0.100
2935 cm^−1^-CH_2_-	0.063	0.023/0.052	0.067/0.054	0.051/0.057	0.054/0.056	0.039/0.052
2853 cm^−1^-CH_3_	0.071	0.078/0.063	0.076/0.066	0.079/0.064	0.073/0.061	0.063/0.055
1730 cm^−1^-C=O	0.090	0.087/0.084	0.104/0.093	0.105/0.094	0.105/0.094	0.107/0.095
1530 cm^−1^ Amide C-N, N-H	0.129	0.124/0.124	0.114/0.124	0.112/0.125	0.115/0.123	0.103/0.121
1310 cm^−1^ Amide N-H	0.043	0.042/0.041	0.042/0.043	0.042/0.045	0.044/0.043	0.044/0.045
1220 cm^−1^ urethane C-O	0.158	0.158/0.165	0.158/0.163	0.159/0.164	0.167/0.167	0.172/0.168
1066 cm^−1^ Soft segment ether bond -C-O-C-	0.341	0.394/0.396	0.348/0.378	0.338/0.375	0.345/0.368	0.309/0.363

**Table 7:** Peak fitting results for carbonyl peaks at 1701 cm^−1^ and 1730 cm^−1^.

Aging Time	HBA of Crosslinked Product	HBA of Uncrosslinked Product
0	/	73%
12	81%	76%
25	85%	76%
50	92%	76%
100	87%	75%
300	82%	75%

## Data Availability

The original contributions presented in this study are included in the article. Further inquiries can be directed to the corresponding authors.
